# Cross-Modal Graph Semantic Communication Assisted by Generative AI in the Metaverse for 6G

**DOI:** 10.34133/research.0342

**Published:** 2024-04-29

**Authors:** Mingkai Chen, Minghao Liu, Congyan Wang, Xingnuo Song, Zhe Zhang, Yannan Xie, Lei Wang

**Affiliations:** ^1^ Key Laboratory of Broadband Wireless Communication and Sensor Network Technology, Nanjing University of Posts and Telecommunications, Nanjing 210003, China.; ^2^ State Key Laboratory of Organic Electronics and Information Displays & Institute of Advanced Materials (IAM), Nanjing University of Posts and Telecommunications, Nanjing 210023, China.

## Abstract

Recently, the development of the Metaverse has become a frontier spotlight, which is an important demonstration of the integration innovation of advanced technologies in the Internet. Moreover, artificial intelligence (AI) and 6G communications will be widely used in our daily lives. However, the effective interactions with the representations of multimodal data among users via 6G communications is the main challenge in the Metaverse. In this work, we introduce an intelligent cross-modal graph semantic communication approach based on generative AI and 3-dimensional (3D) point clouds to improve the diversity of multimodal representations in the Metaverse. Using a graph neural network, multimodal data can be recorded by key semantic features related to the real scenarios. Then, we compress the semantic features using a graph transformer encoder at the transmitter, which can extract the semantic representations through the cross-modal attention mechanisms. Next, we leverage a graph semantic validation mechanism to guarantee the exactness of the overall data at the receiver. Furthermore, we adopt generative AI to regenerate multimodal data in virtual scenarios. Simultaneously, a novel 3D generative reconstruction network is constructed from the 3D point clouds, which can transfer the data from images to 3D models, and we infer the multimodal data into the 3D models to increase realism in virtual scenarios. Finally, the experiment results demonstrate that cross-modal graph semantic communication, assisted by generative AI, has substantial potential for enhancing user interactions in the 6G communications and Metaverse.

## Introduction

Currently, the concept of the Metaverse has attracted both academia and industry. Many companies have begun to focus on the Metaverse [1]. In July 2021, Facebook renamed its company as “Meta”. In addition, Citi expects that the number of Metaverse consumers will exceed 5 billion by 2030, while it will create 8 to 13 trillion dollars in sales. Moreover, it is a higher-dimensional virtual world that is beyond the real world. Therefore, it collects various representations of multimodal data, such as text, audio, video, motion, and touch, from the real world to build a virtual world to provide an immersive experience [2] to consumers. The Metaverse should improve the realism of users’ experiences in the virtual world based on the real world. Thus, the diverse multimodal data should be included in artificial intelligence (AI) [3]. Furthermore, constructing a high-fidelity 3-dimensional (3D) model in a dynamic wireless environment poses significant challenges, remaining a notable concern for 6G communications [4–7].

Recently, with the integration of 6G and AI, semantic communication has emerged [8], which is also considered as a promising technology in 6G. It can directly extract the semantic features in each modality [9] and transmit the intent to the users to achieve efficient interactions between the users. In semantic communication, the current solutions involve deep learning, such as long short-term memory and recurrent neural network, to train multimodal feature vectors [10–12]. However, the convergence of train is often limited, and such methods cannot be applied to the large-scale Metaverse scenarios. In addition, many semantic codecs based on a transformer [13–15] can handle large-scale data efficiently. Since semantic communication contains information abstraction and the representation of semantic features in nature, it is highly suitable for data transmission in the Metaverse.

In addition, the litetures have conducted a lot of research on the semantic communication, mainly focusing on multimodal perception, semantic transmission, and reconstruction. Some of the work on the multimodal perception has focused on the knowledge graphs [16,17] and the alignment in the multimodal data [18] to achieve the semantic association. In the semantic transmission, the authors often design the semantic encoder and decoder to compress the large-scale data [19]. In the reconstruction, the authors often provide the generative adversarial networks (GANs) to quickly reconstruct their spectrum into the new signals [20]. For the Metaverse, the generative reconstruction go through text to 3D or image [21,22]. Most of these works address on the textureless object models [23–25]. Therefore, in order to improve the performance of semantic communication comprehensively, multimodal perception, semantic transmission, and generative reconstruction should be improved at the same time.

Moreover, generative AI approaches can reform the paradigm of semantic communication. Generative AI can construct more realistic content for objects in the virtual world, filling in the diversity of multimodal data. Currently, diffusion models and GANs are mainstream in generative AI. For text and image modalities, the diffusion model [26–28] can learn the denoising process while allowing conditional guidance to flexibly adapt to the semantic reconstruction. The audio and haptic modalities cannot be processed directly so that the authors propose GANs [20,29,30] to reconstruct their spectrum into new signals, quickly [19,31,32]. Hence, generative AI improves efficiency of semantic codec [33–35], accuracy of semantic transmission, and creativity of semantic reconstruction.

As mentioned above, there are several issues that should be considered, such as data bias, semantic ambiguity, channel noise, and illusion generation. Therefore, further verification of the process of transformation and regeneration are urgently needed. Thus, in this article, we present a novel approach, called cross-modal graph semantic communication, to facilitate interactions in the Metaverse. First, in each modality, the graph neural network (GNN) is utilized to mine the attribute expression to find the key information in each object. Second, we propose the utilization of a graph transformer to accomplish cross-modal semantic fusion to increase the ability of the proposed method to prevent interference in wireless communication. Finally, the graph semantic kernel is injected into the generative AI, and the semantic features are employed to drive the rapid reconstruction of the virtual 3D models, which contains the graph relations to describe the details needed to enhance the realism of the 3D models. In contrast to existing semantic communication systems, cross-modal graph semantic communication is more suitable for the Metaverse scenario. It reveals the representation of the multimodal data, associating the semantic kernel and the cross-modal mapping among the different modalities. Moreover, this approach provides a more accurate representations of the 3D models reconstructed by generative AI, resulting in a vivid 3D model that includes multiple modalities.

## Concept

**Fig. 1.**
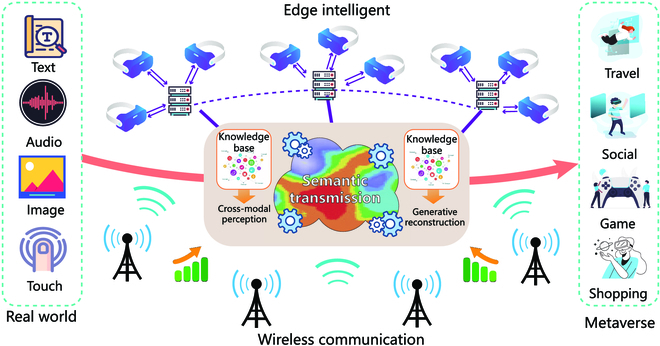
The framework of a cross-modal graph semantic communication system for the Metaverse.

Considering that cognition is a multifaceted and multilayered integration, all kinds of modal signals are the descriptions of the same object in different dimensions. The strong correlations between the important components are inevitably inherent [36], leading to increasingly accurate AI-generated restorations. To better utilize the relationships among the principal components in the modalities, we propose a cross-modal graph semantic communication system. At the transmitter, the semantic kernel of the observed object is visualized as a graph relation. In this way, we can reconstruct the representations in different modalities and break down the barriers caused by inconsistent information representations, which call fill the gap between the virtual 3D models and real data. At the receiver, the potential semantic information is inferred by the modal correlations, and the new features are regenerated by graphs so that we can verify and expand the reconstructed data in the different modalities. The overall system is shown in Fig. 1. Accordingly, we focus on “cross-modal generation” from 3 main aspects: cross-modal perception, semantic transmission, and generative reconstruction in the Metaverse.

In cross-modal perception, we collect text, audio, image, haptic, and other modality data, frequently encountered in the Metaverse. Through AI, such as Transformers and GNNs, we abstract the features of the objects in each modality, individually. Then, the GNN (graph convolutional network) is used to map the different modalities, which we call the feature subgraphs. We aggregate the coefficients of the aligned feature [37–39] by a cross-modal attention mechanism to match every subgraph in a semantic space [16,40,41]. Then, we introduce the graph embedding encoder to extract the semantic feature endogenous relation in each modality. By encoding the graph semantic kernel with the Transformer, we achieve an extreme compression on the key feature information. This approach enables the ultimate dimensional reduction from the data to the features and then to the semantics. Moreover, this approach ensures that the correlations among the multimodal data are not corrupted to achieve a trade-off between efficiency and robustness in cross-modal perception.

In semantic transmission, in contrast to traditional transmissions involving large amounts of data and a high data rate, semantic transmission pays more attention to imbalances. We propose semantic vectors for graph verification to improve cross-modal associations ensuring that semantic information is imbued with the high-fidelity generation while maintaining unbalanced. Our method employs a Transformer decoder with a multihead graph self-attention mechanism and a graph embedding decoder to decode the corrupted semantic vectors into the various constrained correlation subgraphs. Subsequently, the semantic kernel is used to find and correct the error features to obtain the nondistorted subgraphs. We propose a reconstruction of multimodal data guided by a conditional diffusion model to obtain more diversity. Although output of the multimodal data varies, the semantic properties remain unchanged. Hence, this approach alleviates the pressure caused by the large quantity of data in the virtual scenario.

In generative reconstruction, we design a 3D model generation method that combines semantics and multimodal data [42]. We use a 3D generative reconstruction network to drive the large-scale 3D point clouds for semantic analysis [43–45]. We use the generative AI approach to construct a 3D scene and determine the location of each object. Moreover, we use the image modality to fine-tune the distribution of the 3D point clouds and fill in the gaps between the point clouds. Furthermore, we iteratively optimize the surface of the 3D model. From this, we add the generative information from the other modalities, such as audio, text, and haptic information. Thus, the virtual 3D models are rendered with the realistic effects in the modal associations. In this way, we can reconstruct the multimodal data in the Metaverse based on the real world.

## Results and Discussion

### Cross-modal perception

**Fig. 2.**
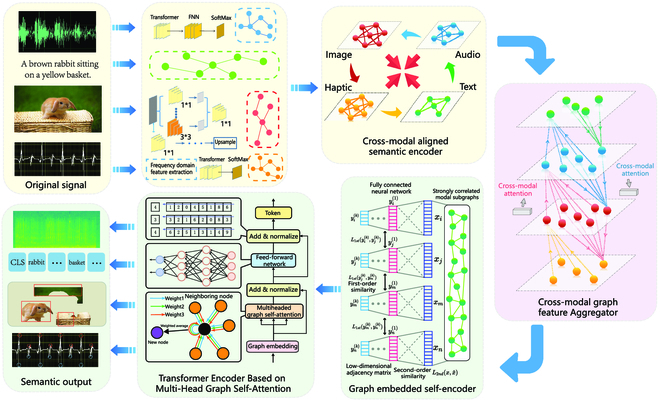
The proposed framework of cross-modal perception. We take a rabbit as an example. The graph features of 4 modal signals about the rabbit (audio, text, image, and haptic) are extracted and aligned by a semantic coder. We aggregate the different modal features of the rabbit through a cross-modal attention mechanism to form a robust relational graph that makes the semantics of the rabbit more complete. The graph structure is compressed by graph embedding, and the semantic features of multimodal rabbit are obtained through the graph Transformer.

As shown in Fig. 2, we condense the key information through cross-modal alignment. Then, we build the subgraphs of the features in each modality, which contain the full semantics. Next, we find each association among all the feature subgraphs to facilitate semantic verification at the decoder. Moreover, the datasets of 4 modalities are constructed into feature subgraphs through the different AI models. We consider the entity and interrelation in the graph as the semantic kernels so that the entities and relations are passed through bidirectional encoder representations from the bidirectional encoder representations from transformers [13,14] to obtain their corresponding vectors. We propose the cosine similarity function to determine the degree of association. We define the relationship of the intramodal feature and further establish the relationship graph of the cross-modal feature. Finally, the semantic kernels in the semantic shared space are condensed. A classifier for cross-modal semantic consistency is constructed from the semantic labels [15]. Moreover, a mechanism for feature verification based on semantic labels is designed to provide a highly robust procedure for semantic compression. Moreover, we further compress the multimodal subgraphs obtained during perception via cross-modal graph semantic compression. The structure of the cross-modal feature graphs will be tighter. Semantic fusion on multiple feature subgraphs is carried out by deep graph embedding based on similarity optimization. Thus, we obtain low-dimensional dense sequences with strong cross-modal semantic associations. Furthermore, we design a semantic vector condensation algorithm that includes a graph Transformer encoder to realize semantic coding on feature graphs.

### Semantic transmission

**Fig. 3.**
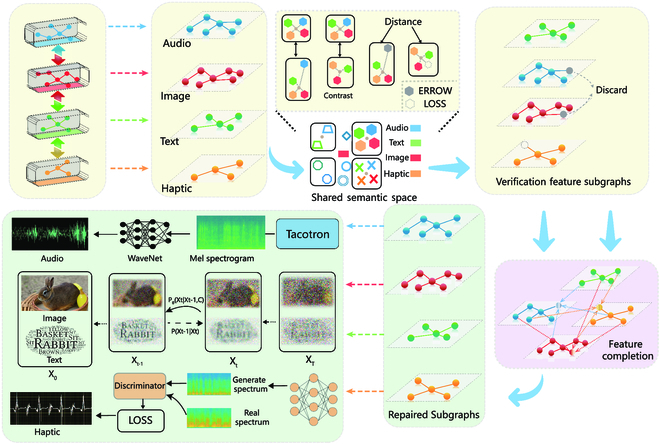
The semantic correction and reconstruction framework can accurately generate the corresponding modal signals. We use distance to verify whether the data are correct. We make corrections via cross-modal correlations to obtain the full semantic graph. Different semantic graphs generate the corresponding reconstructed signals: audio features generate the rabbit’s call through WaveNet, the diffusion model generates a new image of the rabbit and the corresponding prompt of the image, and haptic features generate the haptic analog current signal of the rabbit through GANs. The new data do not restore the initial inputs one-to-one, but they are quite similar meaning in terms of their semantics.

We use cross-modal semantics to drive the reconstruction of multidimensional feature compensation, as shown in Fig. 3. First, we carry out cross-modal subgraph semantic decoding and feature calibration. After the user receives the encoded semantics, the graph transformer is applied to decode the corrupted semantic vectors with the noise. We globally predict the low-dimensional dense sequences using a masked multiattention mechanism and further apply graph embedding to reconstruct cross-modal feature subgraphs. Then, the feature subgraphs are mapped into the shared semantic space. The semantic kernels can verify whether there are erroneous features or missing features under the strong correlation expression to reshape the error and missing features by cross-modal correlation. Moreover, we can refine the cross-modal feature subgraphs.

Thus, we complete the conversion of the low-dimensional vectors to high-dimensional feature subgraphs and further restore the feature subgraphs to the data in each modality through the different generative AI methods. The audio modality takes the form of a Mel spectrogram [46] to generate audio data through the WaveNet vocoder [47]. The haptic modality regenerates data through a GAN. Moreover, the diffusion model is used to regenerate the corresponding text and image data.

We adopt 2 different channel models, additive white Gaussian noise (AWGN) channels and Rayleigh channels, to perform experiments. AWGN is an idealized experimental channel model. Multipath fading is considered in the Rayleigh channel, in which the noise is nonuniform. The 3 modalities used for evaluation are used in several different ways. Regarding the text modality, we map the text data into the semantic vector space by means of the bidirectional encoder representations from transformers. Then, we calculate the cosine similarity of their semantic vectors. For the audio modality, we extract the features from the audio data and evaluate their similarity. With respect to the image modality, we use the similarity to train and calculate the perceptual distance between images. To standardize the criteria, we normalize the similarity results so that the similarity values can be between 0 and 1. A larger value represents greater similarity.

**Fig. 4.**
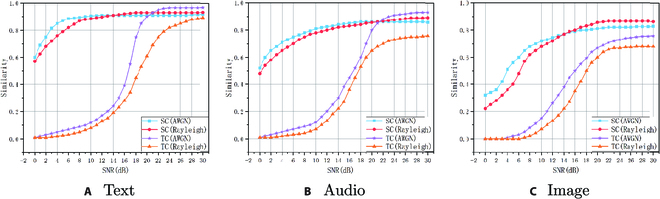
Similarity score versus SNR for the 3 modalities. (A to C) We take 2 comparison variables: one is different channel conditions—AWGN and Rayleigh, and the other is different transmission methods—semantic communication (SC) and traditional communications (TC).

As shown in Fig. 4, the performance on semantic communication is significantly better than traditional communication, especially in the case of a low signal-to-noise ratio (SNR). In the case of a high SNR, the similarity of text and audio when we adopt traditional communication under the AWGN channel slightly exceeds that of semantic communication. For similar images, semantic communication outperforms traditional communication at any SNR. When the SNR exceeds 16 dB, the similarity of all 3 modalities in semantic communication is more than 0.8, and the values of the highest similarity reach 0.93, 0.92, and 0.865, respectively, illustrating the feasibility and superiority of reconstruction via semantic communication.

### Generative reconstruction

**Fig. 5.**
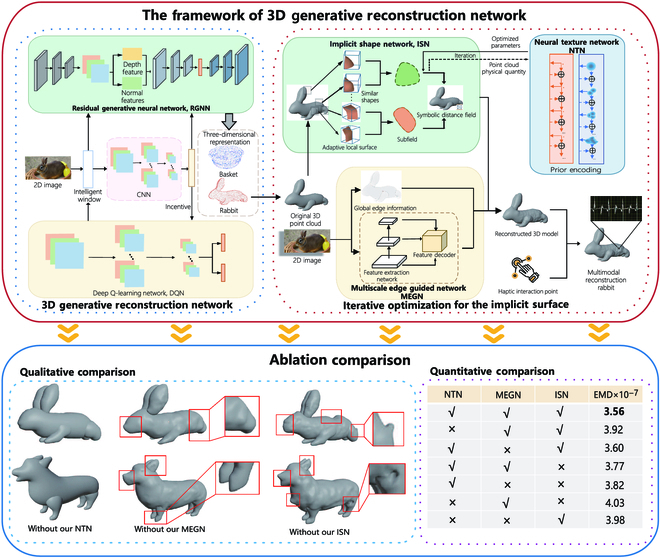
The overall pipeline of the 3D generative reconstruction network. Taking the rabbit as an example, we transferred the picture of the rabbit into a 3D rabbit model, which is quite similar. Through the 3D generative reconstruction network, we can obtain 3D representations of rabbits and baskets from real images. Then, we added the 3D representation texture of the rabbits through the ISN, NTN, and MEGN to describe the details of the rabbit model. Finally, the 3D point clouds are combined with the interaction point of the haptic as the output.

In this article, we design a 3D generative reconstruction network that utilizes intelligent windows that can flexibly change the position and size of objects to detect the environment adaptively [48]. Then, we parse the scene semantics from 2-dimensional (2D) images to 3D point clouds via generative AI reconstruction. As shown in Fig. 5, the 3D generative reconstruction network includes a convolutional neural network (CNN), a deep Q-learning network (DQN), and a residual generative neural network (RGNN). The image pixels containing the intelligent window are sent to the CNN, which automatically learns the features of each object. The CNN obtains the probability of the object through the intelligent window and a 2D excitation vector. The 2D excitation vectors are processed by the DQN, which can find the locations of the objects in the Metaverse. Then, the primary features extracted by the CNN are spliced with the position coordinates. The colors of the objects in the intelligent window are fed into the RGNN. The RGNN further learns the features of the 3D points in the intelligent window. Then, we accurately transform the objects in the intelligent window into a 3D representation. Moreover, we design a data compensation network for the 3D point clouds to refine the representation [49,50]. Because we attempt to combine the main model of the point clouds with the gaps, as in the case of the predicted rabbit ear, a shift network is utilized to further fine-tune the location of the point cloud and eliminate the gaps at the seams.

To further refine the 3D point clouds, we propose an iterative optimization, which consists of the implicit shape network (ISN), the neural texture network (NTN), and the multiscale edge guidance network (MEGN), to address the implicit surface representation issue [51,52]. The workflow of the proposed approach can be found in Fig. 5. The ISN employs a global implicit model to reconstruct the entire surface of the object. The raw point clouds can be turned to a symbolic distance implicit field of the corresponding shape by the ISN [53]. The NTN uses prior encoding to optimize the physical quantities in the implicit functions learned by the ISN. This approach can more accurately reconstruct 3D surfaces and improve the generalizability of the ISN. We also propose the MEGN, which utilizes global edge information and feature representation to endow the reconstructed 3D model with more surface details and textures. Moreover, to increase the multimodal immersive experience, we add interaction points related to haptic, audio, text, and other data to the 3D models. We complete the tasks of multimodal reconstruction such as tactile rendering, voice adaptation, and text filling by detecting changes in interaction points during contact, collision, vibration, and other external surfaces of the 3D models. While the haptic modality is rendered by the unstructured points in a 3D model, we find the location of the haptic interaction points provided by the haptic device corresponding to the force [54,55]. The rendering of other modalities, such as audio and text, is similar to the haptic approach [56,57].

As shown in Fig. 5, 3D point clouds generate from 2D images without the real data of point cloud, so that there is no standard quantitative evaluation benchmark for this method. Therefore, we calculate the Earth Mover’s Distance [58,59] values, which represent the distance between the contour of the side view and the original image, to evaluate the consistency and completeness in the 3D model. In Fig. 5, we also qualitatively present the results of the ablation comparison. It shows that when the framework lacks NTN, the surface of the 3D model is smooth without the texture [60], detail, and shape. When the MEGN is missing, the sketch of the generative model are unclear. For example, the hind legs of the rabbit in the red box stick together and the front legs of the dog are incomplete. When the framework is lack of ISN, the generative model exhibits the unreasonable concavities or protrusions. This experiment effectively proves the effect on NTN. Meanwhile, MEGN can describe the sketchs of the model more clearly. In addition, ISN can make the trend of the surface on the model more reasonable. The entire network including these 3 parts can give the surface on the generated 3D model with more details.

**Fig. 6.**
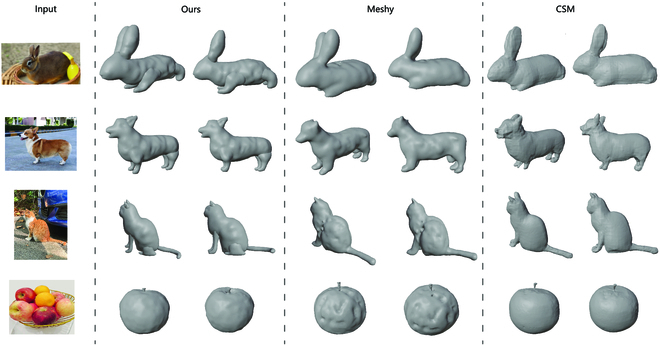
We introduce the images into our proposed method. Then, we compare our method to the Meshy API and CSM. The results show that our method preserves the textural details from different viewpoints. Our method can describe the limbs and tails of different animals, such as the rabbit, dog, and cat, more clearly.

Meanwhile, Fig. 6 shows a qualitative comparison of 3D reconstructions of images from different viewpoints. We introduce the images into our proposed 3D generative reconstruction network and compare them to those of Meshy and CSM. The results indicate that the 3D model in the 3D point clouds obtained by our proposed method is more interpretable and has a more distinct surface than the others. This is because our proposed method includes a refinement network for textures in 3D point clouds, and the generative reconstruction model retains more textures to achieve better effectiveness in 3D models.

## Conclusion

Herein, we propose a cross-modal graph semantic communication system for data interactions in the Metaverse. In this system, we design a multimodal data perception method with a GNN to extract the key information of the object. This approach provides an effective method for decreasing the total quantity of data when enhancing the intramodal and intermodal relationships at the transmitter. Faced with a dynamic environment, noise and channel fading must be considered in data transmission. Therefore, the cross-modal graph semantic communication system addresses on the delivery of semantic information. In addition, this system can integrate multimodal data seamlessly as the same semantic kernel to represent the meaning of an object. Moreover, at the receiver, we also utilize the cross-modal attention mechanism to correct the incorrect semantic information to improve the robustness of the transmission between the users. Based on the characteristics of the different modalities, we adopt different generative AI approaches to accomplish generative reconstruction from semantic features to the representation of multimodal data. Furthermore, according to the image modality, we design a generative network of 3D models by semantics to map 2D image data into the 3D point clouds. Using the intelligent window and several neural networks, we can distinguish various objects in an image, roughly reconstruct the 3D model and visualize certain characteristics, such as the position or relationship between objects. Then, the NTN draws out the details of the texture on the 3D model through multiscale image comparison, which also adds other multimodal data to 3D to make it more vivid. In general, cross-modal graph semantic communication assisted by generative AI provides not only a new perspective on the interaction between the real world and Metaverse but also 3D modeling from a data-driven to a semantic-driven approach, greatly increasing the plasticity of 3D models for the next generation of intelligent systems.

## Data Availability

All the data are available in the manuscript or supplementary materials or from the author.
